# Atrophy Masseter Recovery by Electrical Stimulation Mediated M2‐Like Macrophage Polarisation via JAK/PI3K/AKT Pathway

**DOI:** 10.1002/jcsm.70048

**Published:** 2025-08-15

**Authors:** Chuan Wu, Xiuyun Zheng, Qingchun Li, Yi Chen, Wei Liu, Xinyi Song, Quancheng Han, Qunyan Zhang, Chunfeng Fu, Qing Mei, Xiaoyu Liu, Junji Xu, Jian Zhou, Tingting Wu

**Affiliations:** ^1^ College & Hospital of Stomatology, Anhui Medical University Key Lab. of Oral Diseases Research of Anhui Province Hefei China; ^2^ Department of Periodontics Stomatological Hospital of Chongqing Medical University Chongqing China; ^3^ Salivary Gland Disease Center and Beijing Key Laboratory of Tooth Regeneration and Function Reconstruction, Beijing Laboratory of Oral Health and Beijing Stomatological Hospital Capital Medical University Beijing China; ^4^ Department of VIP Dental Service, School of Stomatology Capital Medical University Beijing China; ^5^ Laboratory for Oral and General Health Integration and Translation, Beijing Tiantan Hospital Capital Medical University Beijing China

**Keywords:** electrical stimulation, macrophage polarisation, masseter muscle atrophy, noninvasive tissue regeneration, PI3K‐Akt

## Abstract

**Background:**

Atrophy of the masseter muscle can result in an aged facial appearance and diminished chewing function. Electrical stimulation (ES) is known for its ability to facilitate tissue healing and functional recovery, but its precise role in the repair of atrophic masseter muscles remains incompletely understood.

**Methods:**

We induced masseter muscle atrophy in rats through botulinum toxin (BTX) injection and subsequently treated the animals with or without ES. Single‐nucleus sequencing (sn‐RNA seq) was conducted to analyse the changes in macrophages of masseter muscles between control, BTX and BTX + ES groups. The role and mechanism of macrophage phenotypic transformation in the process of ES promoting the recovery of atrophied masseter muscles were both verified through in vivo and in vitro experiments.

**Results:**

Our results indicate that ES treatment within defined current parameters significantly ameliorated muscle condition by reducing atrophy‐related gene expression (*MuRF1*: BTX: 10.15 ± 1.69; BTX + ES: 1.05 ± 0.06; *Fbxo32*: BTX: 8.62 ± 1.19, BTX + ES: 1.19 ± 0.07, *p* < 0.0001) and enhancing vascularisation (Vegf positive area: BTX: 6.60 ± 2.87%, BTX + ES: 27.23 ± 1.70%, *p* < 0.001). Analysis conducted with sn‐RNA seq demonstrated increased infiltration of M1 macrophages during muscle atrophy, with a subsequent transition to M2 macrophages following ES treatment (M1 macrophage portion: Ctrl: 15.2%, BTX: 25.8%, BTX + ES: 14.7%; M2 macrophages: Ctrl: 67.9%, BTX: 46.9%, BTX + ES: 70.5%). Further investigations demonstrated that ES reduced M1 macrophage infiltration (five‐fold lower of CD86^+^ cell number, BTX: 30 ± 2; BTX + ES: 6 ± 2, *p* < 0.0001) while increasing M2 macrophage presence (3.3‐fold higher of CD163^+^cell, BTX: 10 ± 3; BTX + ES: 33 ± 8, *p* < 0.01), potentially via activation of the PI3K‐Akt pathway (p‐Akt/Akt ratio, BTX:0.58 ± 0.20%; BTX + ES:1.03 ± 0.07%, *p* < 0.05). Depletion of macrophages using clodronate liposomes reversed the beneficial effects of ES on induced masseter atrophy (*MuRF1*: BTX + ES: 2.20 ± 0.16; BTX + ES + CL: 12.93 ± 0.98, *p* < 0.0001), highlighting the involvement of macrophages in the therapeutic process. In vitro studies demonstrated that ES promoted the transition from M1 to M2 macrophages and enhanced proliferation and differentiation of myogenic cells.

**Conclusions:**

Our findings suggest that ES can enhance masseter muscle tissue repair by modulating macrophage polarisation, offering valuable insights into the potential of ES in noninvasive tissue regeneration strategies for treating masseter muscle atrophy.

## 1 Introduction

The skeletal muscle is crucial for maintaining glucose and lipid homeostasis, powering the motor system and serving as a significant protein reservoir in the body. Similarly, the masticatory muscle, including the masseter, temporalis, lateral pterygoid and medial pterygoid muscles, has a notable impact on craniofacial morphology and masticatory function [1]. The prevalence of masticatory muscle atrophy is increasing, with mild atrophy leading to temporal or cheek drooping and compromised lower jaw function, while severe atrophy can significantly impair mastication and overall quality of life.

While muscle tissue possesses a high capacity for self‐repair following injury, severe injuries resulting in substantial muscle loss can lead to permanent functional impairments. Therefore, interventions for muscle regeneration and repair are essential and often necessary. Common modalities for addressing muscular dystrophy include physical methods such as resistance training, mechanical loading, shock waves, vibration waves and lasers [2, 3, 4, 5]. Electrical stimulation (ES) has emerged as a promising noninvasive therapeutic approach for combating muscle atrophy in various contexts [5, 6]. ES is increasingly being used as an alternative or complementary therapy for injured muscle tissue to enhance blood flow, promote mitochondrial biogenesis and reduce inflammation during musculoskeletal tissue rehabilitation [7, 8, 9]. Recent research has demonstrated that ES can lead to a 1% increase in muscle mass and a 10%–15% improvement in muscle function after 5–6 weeks of stimulation [10]. It has been observed that growth factors released by adjacent epithelial cells play a significant role in the proliferation and differentiation of muscle satellite cells, thereby facilitating the regeneration and maturation of muscle fibres and promoting angiogenesis [11]. However, the diversity in methodologies, devices and ES parameters utilised by different researchers hinders direct comparisons and definitive conclusions on its efficacy [12, 13].

Furthermore, despite the anatomical and biochemical distinctions between masticatory muscles and those of the trunk and limbs [14, 15, 16], the majority of studies have predominantly focused on skeletal muscles in the lower extremities, with limited exploration of the mechanisms underlying masticatory muscles or corresponding treatment modalities [6, 17]. Previous studies have indicated that cranial muscles exhibit a broader range of contractile protein expression and function compared to other skeletal muscles, with certain type I fibres in the masseter muscle displaying velocities 10 times slower than type I fibres in limb muscles.

A complex interplay occurs between muscle satellite cells and the innate immune system, involving neutrophils, macrophages and dendritic cells, within a non‐visualised environment [18]. These immune cells play crucial roles in eliminating damaged muscle fibres, releasing specific cytokines to regulate the activation and differentiation processes of satellite cells and directing adjacent stromal cells to participate in angiogenesis and extracellular matrix (ECM) remodelling. Specific subpopulations of macrophages have been shown to create a temporary niche for stem cell proliferation at the injury site, facilitating skeletal muscle regeneration [19]. Macrophages play dual pro‐ and anti‐inflammatory roles in muscle atrophy recovery. Initially, M1 macrophages orchestrate inflammation by releasing various cytokines and chemokines [20]. Subsequently, M2 macrophages secrete anti‐inflammatory molecules and anabolic growth factors. However, the precise contribution of macrophages to masticatory muscle regeneration through ES remains unclear.

In this study, our objective was to explore the reparative and regenerative effects of ES on the masseter muscle, including its associated blood vessels and nerves, in a rat model of masseter muscle atrophy. To delve further into the underlying immune mechanisms, single‐nucleus RNA sequencing (sn‐RNA seq) was utilised to analyse the cellular composition of healthy and atrophied masseter muscles, focusing on macrophage polarisation dynamics during ES treatment.

## 2 Methods

### 2.1 Animals

Six‐week‐old male Sprague–Dawley rats, weighing 180–200 g, were purchased from the Experimental Animal Center of Anhui Medical University. They were housed under specific pathogen‐free conditions (20 ± 2 °C, 12 h light/dark cycle, 48%–50% humidity) with free access to food and water. The study protocol was approved by the Institutional Animal Care and Use Committee of Anhui Medical University (Permit No. LLSC20221123), and the animals' care was in accordance with institutional guidelines.

### 2.2 Masseter Atrophy Model and Electrical Stimulation Treatment

The rats in the Ctrl group were injected with normal saline on both sides of the masseter muscle (60 μL). Each rat in the experimental group received a bilateral intramuscular injection of BTX with the same volume of saline solution (1.5 U; 60 μL; Hengli, Lanzhou, China) [21]. Doses and volumes were determined by LD50 [22] and mean body weight according to previous studies [23]. The intramuscular injection points are the centre points of the superficial and deep masseter muscle [24]. The details are described as follows: The superficial intramuscular injection point is located at the vertical line passing through the midpoint of the line between the lateral orbital canthus and the external auditory canal orifice at the intersection with the line connecting the mandibular angle and the external oral cavity. The deep intramuscular injection site is located just below the lateral orbital canthus and 1–2 mm below the zygomatic bone (Figure S1a). To fix the injection, we placed a plastic stopper on the needle blade.

ES device was used to deliver stimulation to the masseter muscle through a sheet of electrodes that can be clamped on the skin as a noninvasive therapeutic tool. This device (Jiajian. China, CMNS6‐2) can provide the intermittent wave: The frequency is continuously adjustable from 1 to 100 Hz; the break wave time was 5 s ± 2 s, and the continuation wave time was 15 s ± 3 s (Figure S1a). First, the appropriate voltage was selected as 20 V, and the frequency was selected as a lower 10 Hz and a higher 50 Hz for comparison according to previous studies [10]. Second, to determine the current intensity, the rats were immobilised to receive ES treatments without anaesthesia to test the optimal range that causes rhythmic vibration of the masseter but did not allow the rats to resist violently. Based on dose–response studies (5–8 mA), 5–7 mA was selected as the optimal intensity, as it did not cause pain‐related stress (e.g., vocalisation or escape behaviour). Higher currents (e.g., 8 mA) triggered adverse responses and were excluded. Finally, rats in the experimental group were treated with ES of six different parameters (5 mA/10 Hz, 5 mA/50 Hz, 6 mA/10 Hz, 6 mA/50 Hz, 7 mA/10 Hz, 7 mA/50 Hz). ES treatment started 1 day after BTX injection and was carried out with 2 s of stimulation followed by a 6‐s rest. ES was conducted for 20 min a day, six times a week and was performed at the area of the skin surface corresponding to the inner and outer ends of the masseter muscle (Figure S1a). ES was performed under isoflurane anaesthesia (2%–2.5%) to eliminate stress‐induced movement artefacts and ensure stimulation precision. All experimental groups (including non‐stimulated controls and BTX group) received identical anaesthetic exposure to maintain procedural consistency. Animals were sacrificed by euthanasia after 1, 3, 7 and 14 days of intervention.

### 2.3 The use of PI3K Inhibitors

LY294002 (MCE, China) was administered via intramuscular injection. As a specific PI3K inhibitor, LY294002 suppresses PI3K/Akt signalling activity. Rats received daily intramuscular injections at a dose of 1.5 mg/kg/day for 14 consecutive days [25]. Control rats were administered an equivalent volume of PBS under identical conditions.

### 2.4 Macrophage Depletion Study in Vivo

The rats in the experimental groups were randomised and divided into three groups: (1) PBS‐liposome treated group (Ctrl); (2) PBS‐liposome, BTX and ES‐treated group (BTX + PBS + ES); and (3) clodronate‐liposome (CL), BTX and ES‐treated group (BTX + CL + ES). The methods of macrophage depletion were described in a previous study [2]. For the liposome‐treated groups, 100 μL of PBS‐ or clodronate‐containing liposomes was administered via tail vein injection a day before BTX injection and again on Days 2, 5 and 8. An additional 10 μL of PBS‐ or clodronate‐containing liposomes was also administered intramuscularly by injection at the centre of the masseter on Days 2, 5 and 8 after BTX injection. After 14 days, the masseter muscle was isolated for analysis.

### 2.5 snRNA‐Seq and Bioinformatics Analyses

Nuclei were purified and were loaded into the 10 × Chromium Chip. snRNA‐seq libraries were prepared using the Chromium Single Cell 3 Reagent Kits v3 and sequenced on the Illumina HiSeq4000 platform. Cell Ranger (V6.0) was used to map reads to the reference genome and acquire gene counts to generate expression matrix files for subsequent analysis. Various R packages and software were used, including Seurat (V4.1.1) to filter data; Wilcox algorithm to identify marker gene, ClusterProfile for KEGG pathway enrichment analysis; javaGSEA for gene set enrichment analysis; Monocle3 for trajectory analysis. The sequencing service were provided by Personal Biotechnology Co. Ltd. Shanghai, China.

### 2.6 RAW 264.7 and Cell Electric Stimulation

The RAW 264.7 cell line is a widely used murine macrophage model. Cells were cultured in RPMI 1640 medium supplemented with 10% (v/v) foetal bovine serum (FBS) (Gibco, Alcobendas, Spain) and 1% (v/v) Penicillin/Streptomycin (Sigma‐Aldrich, Madrid, Spain). Cells were seeded on 1 mL of completed medium in each of the 24 wells at a cellular density of 100 000 cells. RAW264.7 cells were stimulated with LPS (1ug/mL) for 12 h to prepare M1 macrophages. After successful LPS induction, the medium is replaced with serum‐free culture medium, and then, ES is applied with parameters of 2 mA current, 2 V voltage and a stimulation duration of 30 min per day. After continuous stimulation for 3 days, the cells are then co‐cultured in a chamber containing C2C12 cells, and the medium is replaced with C2C12 culture or myotube differentiation induction medium (as described in supplemental material C2C12). Cells were maintained at 37°C in a humidified atmosphere with 5% CO_2_.

### 2.7 Statistical Analysis

Data were analysed using the GraphPad Prism 6 software (USA). Student's *t* tests were used to compare parametric data between two groups, and one‐way or two‐way analysis of variance (ANOVA) was used to analyse multiple conditions that were more than two groups. Post hoc pairwise comparisons were determined by Tukey's test. *p* < 0.05 was considered statistically significant.

## 3 Results

### 3.1 ES Promotes the Recovery of Atrophied Masticatory Muscles With the Restoration of Blood Vessels and Nerves

**FIGURE 1:**
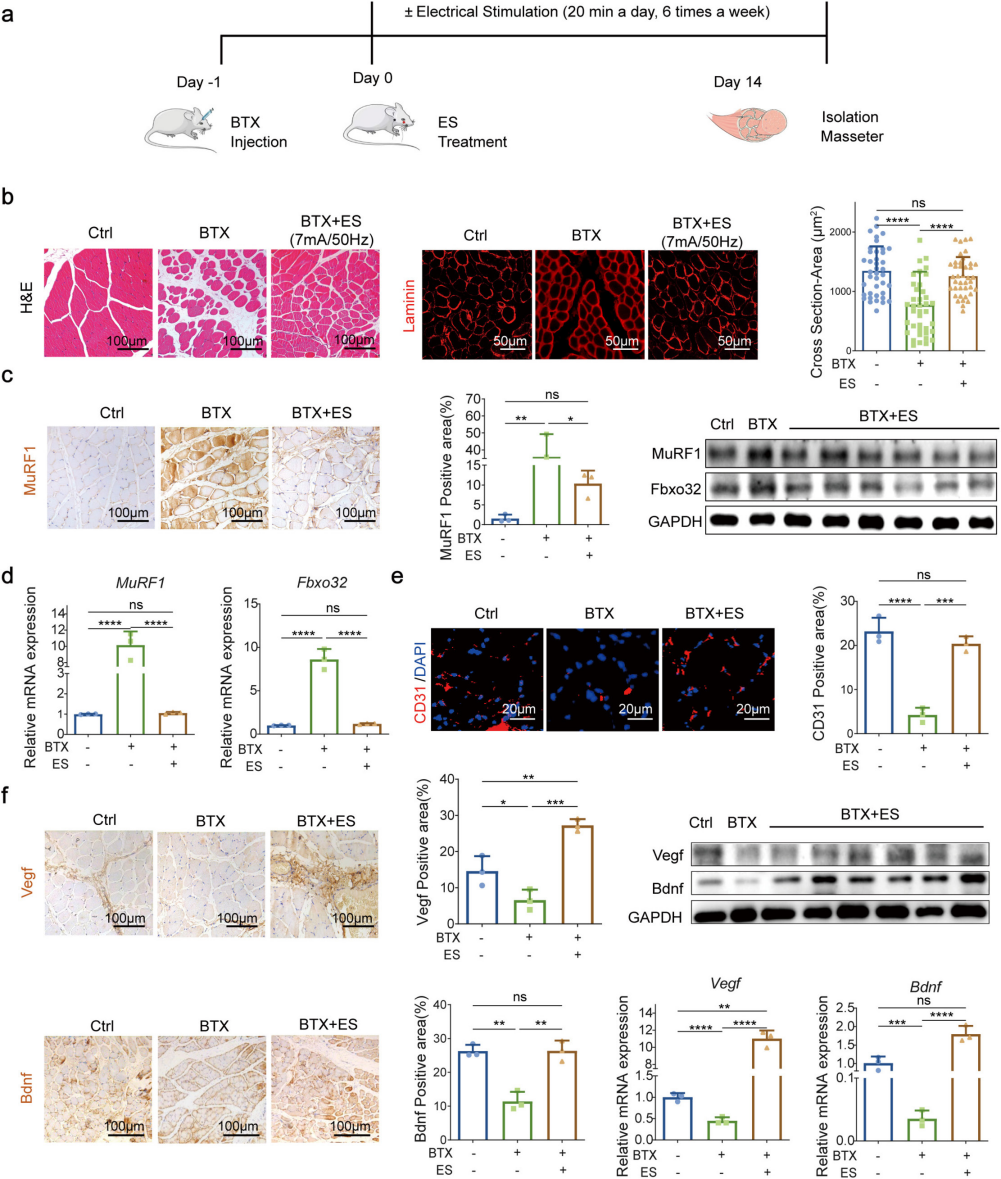
ES promotes the recovery of atrophied masticatory muscles with the restoration of blood vessels and nerves. (a) A schematic diagram. The masseter muscle of rats was injected with BTX, and then, ES device was used to deliver stimulation. Samples were obtained on day 14 for testing. (b) H&E stain of cross sections of masseter muscles. Scale bars, 100 μm. Representative IF images of laminin. Scale bars, 50 μm. Cross‐sectional areas of muscle fibres. (c) IHC images of MuRF1. Scale bars, 100 μm; MuRF1 positive area; WB analysis of MuRF1 and Fbxo32. (d) qRT‐PCR analysis of *MuRF1* and *Fbxo32* mRNA. (e) Representative IF images of CD31 (red) and DAPI (blue) staining. Scale bars, 20 μm; CD31 positive area (right). (f) Representative IHC images of Vegf and Bdnf. Scale bars, 100 μm. Quantification of positive regions of Vegf and Bdnf; WB analysis of Vegf and Bdnf; qRT‐PCR analysis of *Vegf* and *Bdnf*. All data represent three independent experiments (*n* = 3). Error bars indicate SD; *p < 0.05, **p < 0.01, ***p < 0.001, ****p < 0.0001.

This study aimed to investigate therapeutic regimens and mechanisms utilising noninvasive ES for atrophic masticatory muscles. BTX often acts on the neuromuscular junction, causing muscle relaxation and atrophy by interfering with the transmission of nerve‐muscle signals [26]. To reliably induce masseter muscle atrophy, BTX was administered into the masseter muscle of rats [27]. On the second day, ES therapy was initiated by clamping the electrode piece of the ES devices onto the skin surface of rats, aligning with the ends of the long axis of the masseter muscle. Past studies have suggested that ES systems can effectively address skeletal muscle atrophy when applied at frequencies ranging from 10 to 50 Hz, voltages ranging from 10 to 30 V [10, 28] and currents ranging from 4 to 16 mA. Initial investigations showed that stimulation intensities below 5 mA/10 Hz did not exhibit a reduction effect in atrophy compared to an untreated group (data not shown). When the intensity reached 8 mA, rats exhibited marked discomfort and resistance, making it challenging to use higher intensities in these studies. Consequently, subsequent experiments focused on the intensity range of 5 mA/10 Hz to 7 mA/50 Hz. Six different stimulus intensities (5 mA/10 Hz, 5 mA/50 Hz, 6 mA/10 Hz, 6 mA/50 Hz, 7 mA/10 Hz, 7 mA/50 Hz) were applied. The therapy continued for 2 weeks. In contrast, rats in the negative control (Ctrl) group did not receive any treatment and rats in the BTX group were treated with BTX injections without ES (Figure 1a; S1a).

After 2 weeks, the masseter muscles were isolated. The shape of the right masseter muscles in rats injected with BTX was notably smaller, but it gradually recovered as the intensity of ES increased (Figure S1b). Importantly, the intensity of stimulation remained within a tolerable range and did not cause harm to the rats or affect their normal daily activities. As a result, there was no significant difference in the body weight of the rats in each group (Figure S1c). The results of H‐E staining and Laminin staining demonstrated that the muscle fibres were irregularly condensed, but the extracellular space was expanded in the BTX group, with reduction of the cross‐sectional area. After the application of the six different ES regimens, orderly arrangement of muscle fibres was observed, with a significant reduction in damaged muscle fibres in the ES group. Based on the significant improvements observed with ES at 7 mA/10 Hz or 7 mA/50 Hz in the previous experiments, either of these intensities can be considered optimal (Figure 1b; S1d,e). For the subsequent experiment, a stimulation intensity of 7 mA and a frequency of 50 Hz was selected. Furthermore, to evaluate whether the histological improvements mediated by ES correlated with the recovery of muscle strength, the marker genes related to muscle atrophy, such as MuRF1 and Fbxo32, were measured. These genes are known to be upregulated in atrophied muscles. It showed that the expression of MuRF1 in the masseter was significantly increased in the BTX injection group but decreased after ES. To further confirm the recovery of muscles, western blot assays were applied. The expression of MuRF1 and Fbxo32 was decreased in the ES group (Figure 1c). The relative expression of ***MuRF1*** and *Fbxo32* mRNA in the ES group showed a continuous decrease, which was consistent with the trend of protein expression (Figure 1d; S1f), suggesting that ES accelerates the rate of tissue regeneration. ES has been recognised for its potential in improving the rehabilitation of musculoskeletal tissues, potentially by enhancing blood flow [29].

Given the critical role of neurovascular‐related factors in the repair of atrophic muscles, they contribute as supplementary indicators for monitoring muscle regeneration outcomes. Initially, the expression of angiogenesis‐related cytokines, CD31 and Vegf, was assessed using immunofluorescence (IF) and immunohistochemistry (IHC) assays. Following BTX injection, the expression of CD31 declined, while ES treatment led to an increase in CD31 expression (Figure 1e). Similarly, the intensity of Vegf‐positive immunostaining and the protein expression of Vegf exhibited a comparable expression pattern, suggesting that BTX suppressed angiogenesis, while ES promoted regeneration in masseter tissue. Moreover, Bdnf, a classic peptide neurotransmitter, is involved in muscle regeneration [30]. The expression of Bdnf was increased in masseter after ES as determined by the IHC assay. Then, the protein expression of Bdnf was detected by western blot assays. They were all upregulated in the ES‐treated groups. In addition, the mRNA levels of *Vegf* and *Bdnf* were also upregulated in masseter with ES treatment (Figure 1f; S1f). All these results indicated that ES can effectively improve the rehabilitation of atrophic masticatory muscles.

### 3.2 snRNA‐seq Revealed That ES Promotes the Polarisation of M1 Macrophages Toward the M2 Phenotype in Atrophic Masseter Muscles

**FIGURE 2:**
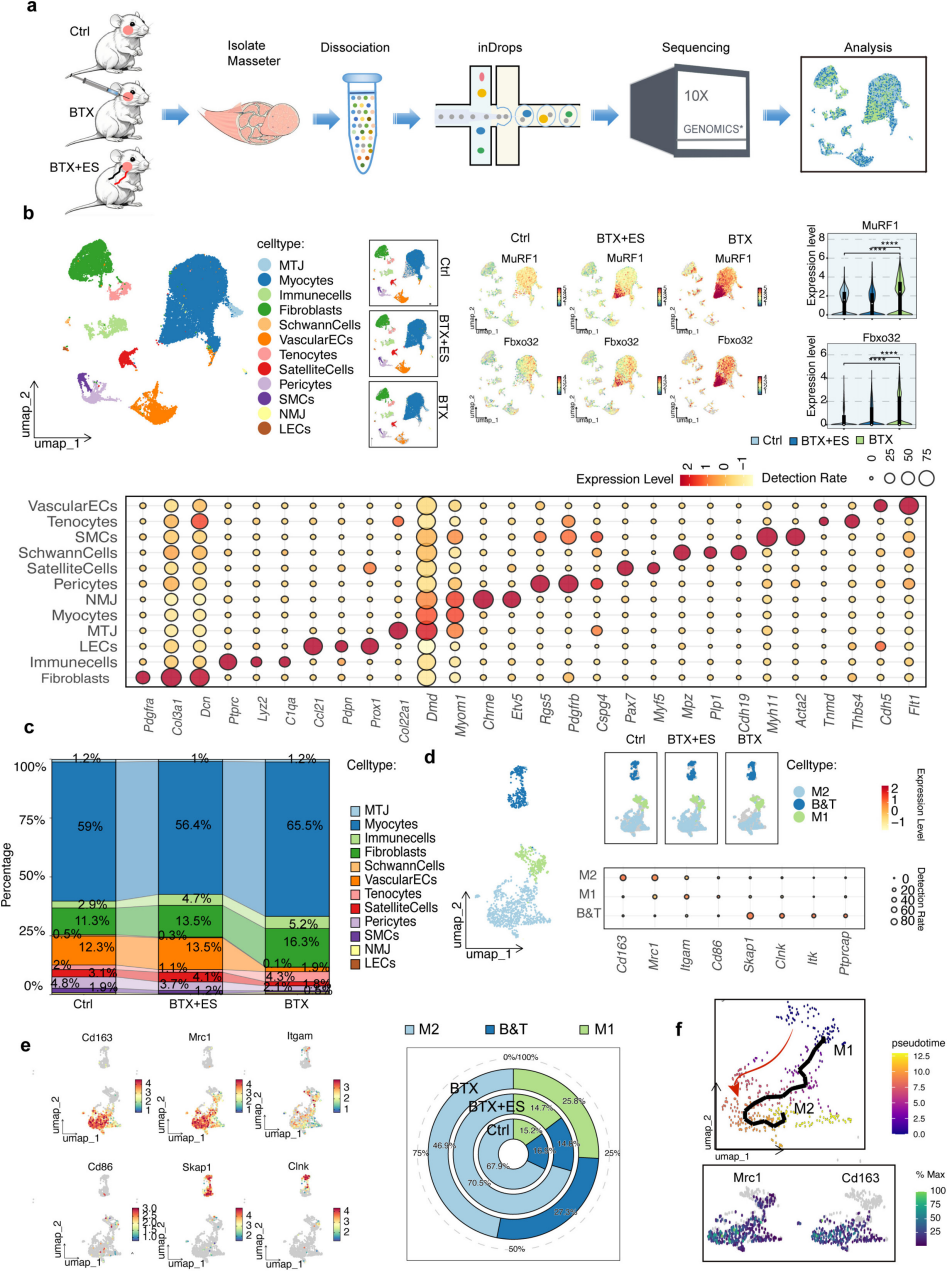
sn‐RNA seq revealed that ES promotes the polarisation of M1 macrophages toward the M2 phenotype in atrophic masseter muscles. (a) Schematic diagram of sn‐RNA seq. (b) UMAP plots were colour‐coded according to the expression of marker genes of all cells from the Ctrl, BTX, and BTX+ES samples. Dot plots according to the expression of marker genes based on different cells. (c) Analysis of cell fractions within different cell groups. (d) UMAP plots of Immune cells (left); Dot plots according to the expression of marker genes based on different immune cells (right). (e) Feature plot of different marker genes (left); analysis of cell fractions within different immune cells (right). (f) Pseudotime analysis of macrophages. Error bars indicate SD, *p < 0.05, **p < 0.01, ***p < 0.001, ****p < 0.0001.

Previous studies have shown that muscle atrophy is accompanied by some changes in the local microenvironment, for example, the alteration of inflammatory factors and the occurrence of cell apoptosis, which can also trigger immune responses [18]. Therefore, we investigated the levels of inflammatory factors in the atrophied masseter muscle induced by BTX and found the mRNA expression levels of the inflammatory‐related factors, such as *Tnf‐α* and *IL‐1β* increased (Figure S2a,b). Additionally, it was observed that the number of apoptotic cells increased after BTX‐induced atrophy using TUNEL staining (Figure S2c). To further elucidate the interplay between masseter atrophy and the immune microenvironment, and to investigate whether ES modulates this milieu to influence tissue repair, we employed the 10 × Genomics Chromium platform to profile the heterogeneity of immune cell responses through snRNA‐seq. Then, the single‐nucleus libraries were created, and subsequently, sequencing was performed (Figure 2a). After excluding cells of subpar sample quality, we retained a total of 63 530 nuclei for further analysis. Utilising UMAP, we conducted initial visualisation and two‐dimensional mapping, which resulted in the identification of 31 unsupervised clusters by snRNA‐seq (Figure S3a). Based on the expression of lineage‐specific markers, we consolidated redundant cell clusters and assigned them to 12 main cell populations. These populations include myotendinous junction (MTJ), neuro muscle junction (NMJ), tenocytes, satellite cells, smooth muscle cells (SMCs), pericytes, lymphatic endothelial cells (LECs), vascular endothelial cells (ECs), fibroblasts, immune cells, Schwann cells and myocytes (Figure 2b,c). Furthermore, the increase in ***MuRF1*** and *Fox32* expression was significantly more pronounced in the BTX group than in the ES group (*p* < 0.05) (Figure 2b). Analysis of cell fractions within each group revealed an increase in the number of immune cells in both the BTX and BTX + ES groups, suggesting its potential role in muscle atrophy (Figure 2c). To further examine the dynamics of immune cell subtypes following BTX injection and ES treatment, we conducted sub‐clustering analysis using the background of the snRNA‐seq data. This analysis resulted in the identification of seven subclusters (Figure S3b). By assessing the expression of specific markers (Figure 2d,e), we classified these subclusters into three main cell populations: B/T cells, M1 macrophages and M2 macrophages, with macrophages being the most abundant. To further analyse changes in different macrophage subtypes, we examined the proportions of each cell type. We observed that compared to the control group, the proportion of M1 macrophages significantly increased while the proportion of M2 macrophages decreased in the BTX group. Following ES treatment, the proportion of M2 macrophages increased and the proportion of M1 macrophages decreased, suggesting that ES may promote the conversion of M1 to M2 macrophages. To further validate this result, we performed pseudotime analysis on macrophages in the ES group. The analysis revealed a tendency for M1 macrophages to transition toward the M2 phenotype in the ES group, accompanied by an increase in M2‐associated markers *Mrc1* and *Cd163* (Figure 2f). Along the pseudo‐temporal process, the gene expression associated with muscle regeneration was increased, such as *Kcnt2* and *Scl9a9* (Figure S3e). This suggests that macrophage polarisation may play an important role in muscle atrophy and regeneration. Additionally, cellular communication analysis indicated close communication between M1 macrophages and M2 macrophages, which indicates that macrophage polarisation may be involved in the process of the masseter muscle recovery stimulated by ES (Figure S3f).

### 3.3 ES Promotes the Transition From M1 to M2 Macrophages Though PI3K‐Akt Pathway

**FIGURE 3:**
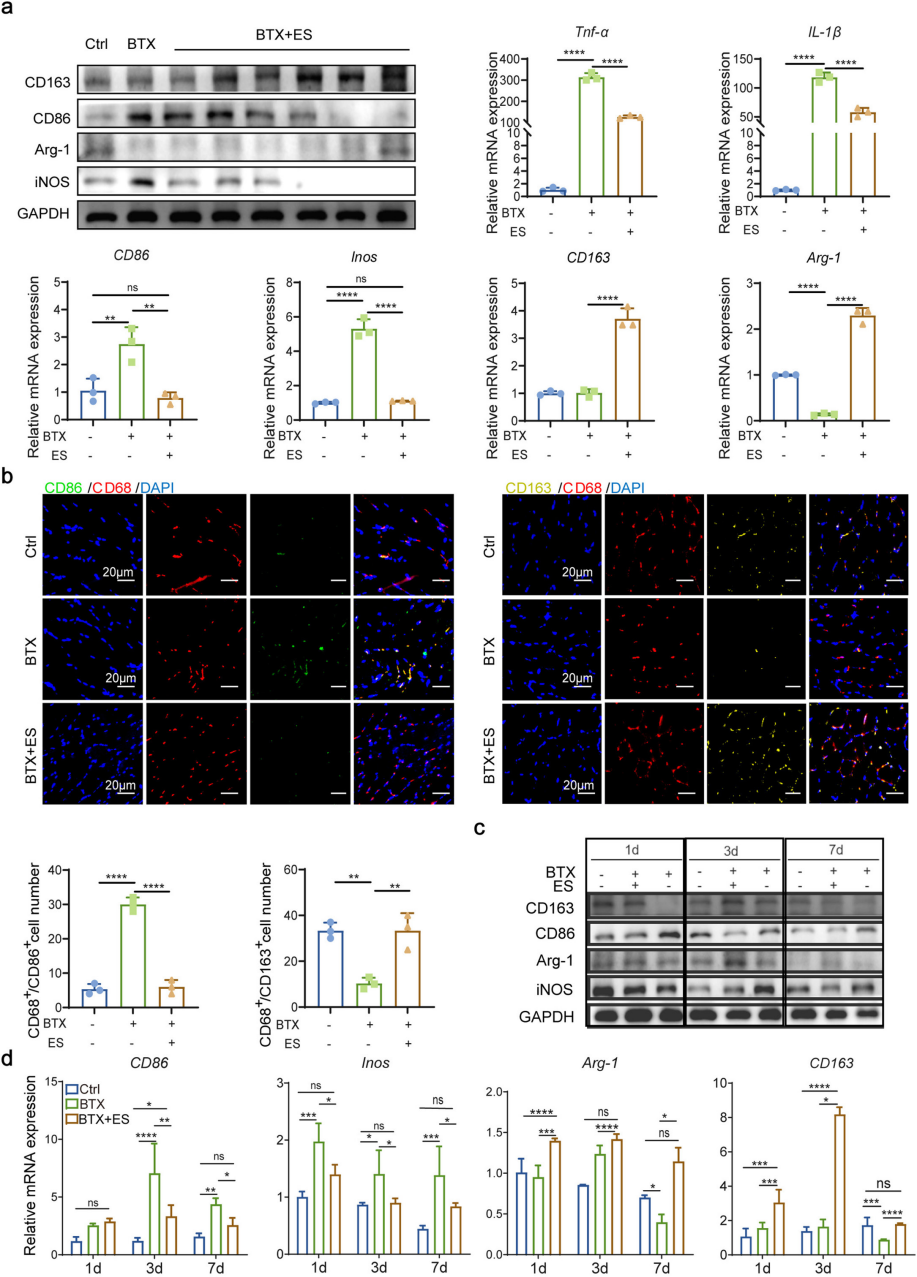
Electrical stimulation promotes the polarization of M2‐type macrophages during the recovery process of atrophy masseter muscle. (a) WB analysis of CD86, iNOS, CD163 and Arg‐1; qRT–PCR analysis of *Tnf‐α*, *IL‐1β*, *CD86*, *Inos*, *CD163* and *Arg‐1*. (b) Representative IF images of CD68 (red), CD86 (green), and DAPI (blue). Representative IF images of CD68 (red), CD163 (yellow) and DAPI (blue). Scale bars, 20 μm; the number of CD68^+^/CD86^+^ cells and CD68^+^/CD163^+^ cells. (c) WB analysis of CD86, iNOS, CD163 and Arg‐1 and quantification of relative band intensity. (d) qRT–PCR analysis of *CD86*, *Inos*, *CD163* and *Arg‐1* mRNA. All data represent three independent experiments (*n* = 3). Error bars indicate SD; *p < 0.05, **p < 0.01, ***p < 0.001, ****p < 0.0001.

To further validate the role of macrophage polarisation in the recovery of masseter muscle atrophy, the atrophic masseter muscle was treated with ES and the expression of marker‐related genes to macrophage polarisation was assessed. After 14 days of ES treatment, it was found that the expression levels of CD86 and iNOS proteins associated with M1 macrophage polarisation decreased in the ES group compared to those in the untreated BTX group. Conversely, the levels of proteins associated with M2 macrophage polarisation, such as CD163 and Arg‐1, increased after ES stimulation (Figure 3a; S34). The mRNA expression of M1 macrophage polarisation–related genes like *Tnf‐α*, *IL‐1β*, *CD86* and *Inos* increased after the induction of muscle atrophy by BTX, whereas genes associated with M2 macrophages like *CD163* and *Arg‐1* decreased. However, the expression of M1 macrophage‐related genes decreased, while the expression of M2 macrophage‐related genes increased after ES (Figure 3a). To locate M1 and M2 macrophages, IF staining assay was performed. The number of CD68^+^/CD86^+^ cells, indicating M1 macrophages, increased after BTX injection but decreased after ES treatment. Conversely, the number of CD68^+^/CD163^+^ cells, indicating M2 macrophages, showed the opposite expression trend (Figure 3b).

We further investigated whether the changes related to macrophage polarisation were time‐dependent. It demonstrated that the expression of CD163 and Arg‐1 reached its peak on the third day and gradually decreased thereafter, and it remained higher than that in the BTX group. Interestingly, there was a transient increase in M1 macrophages 1–3 days after BTX injection. Despite the overall decrease in the expression intensity of CD86 and iNOS from 3 to 7 days, their expression levels were still higher in the BTX group compared to the ES group (Figure 3c; S3f). Additionally, the mRNA expression of specific macrophage subtypes was examined. The ES groups of masseter tissue exhibited similar expression trends of proinflammatory M1 macrophages and pro‐healing M2 macrophages compared to Ctrl mice at both time points (Figure 3d). These findings indicate that ES can potentially reduce the polarisation of M1 macrophages and promote their transition into M2 macrophages during the early stage in the recovery of masticatory muscles.

**FIGURE 4:**
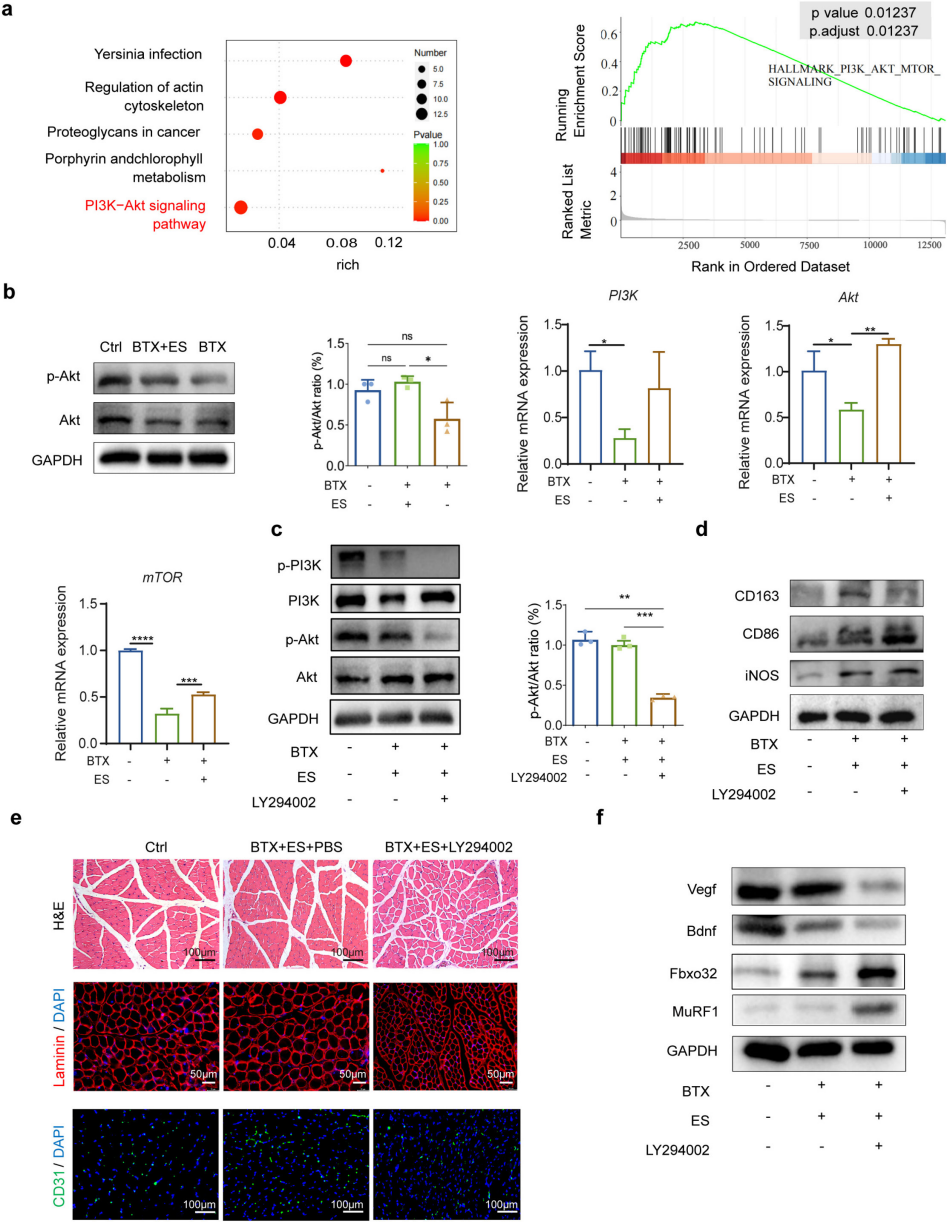
ES promotes the transition from M1 to M2 macrophages though PI3K‐Akt pathway. (a) KEGG analysis of M2 macrophages; GSEA analysis of PI3K‐Akt pathway in the Ctrl group compared to the BTX group. (b) WB analysis of p‐Akt and Akt and quantification of relative band intensity; qRT–PCR analysis of *PI3K, Akt* and *mTOR*. (c–f) Samples from rats in different groups: control, negative control (BTX + ES + PBS), and BTX + ES + LY294002 (LY294002: inhibition of PI3K‐Akt pathway). (c) WB analysis of p‐Akt, Akt, p‐PI3K, and PI3K and quantification of relative band intensity. (d) WB analysis of CD163, CD86 and iNOS. (e) H&E stain of cross sections of masseter muscles (top). Scale bars, 100 μm; representative IF images of laminin (middle); scale bars, 50 μm; and representative IF images of CD31, Scale bars, 100 μm. (f) WB analysis of Vegf, Bdnf, Fbxo32 and MuRF1. All data represent three independent experiments (*n* = 3). Error bars indicate SD, *p < 0.05, **p < 0.01, ***p < 0.001, ****p < 0.0001.

To further investigate the mechanism underlying the transformation from M1 to M2 macrophages, the KEGG analysis of macrophages was conducted. The result indicated significant enrichment of the PI3K‐Akt pathway. The GSEA results revealed that this pathway was activated in the Ctrl group compared to that in the BTX group, suggesting its suppression after masseter muscle atrophy (Figure 4a). Consistent with these findings, the protein expression of p‐Akt increased in the ES groups compared to those in the BTX group, with the downregulation of mRNA expression of *PI3K*, *Akt* and *mTOR* (Figure 4b), indicating the activation of this pathway during ES treatment. To validate the findings, we administered the PI3K inhibitor LY294002 to inhibit the PI3K‐Akt pathway. As a result, there was a decrease in the phosphorylation levels of Pi3k and downstream gene Akt (Figure 4c; S4a). Additionally, the verification of macrophage polarisation–associated proteins revealed an increase in CD86 and iNOS (M1) and a decrease in CD163 (M2) (Figure 4d; S4a), indicating that the process of ES promoting macrophage polarisation from M1 to M2 macrophages has been inhibited. Furthermore, the effectiveness of ES in promoting the recovery of atrophic masseter muscle was also inhibited, with no decrease in atrophy masseter–related gene expression like MuRF1 and Fbxo32 and no increase in angiogenesis‐related protein expression, such as CD31, Vegf and Bdnf (Figure 4e–f; S4b).

Previous studies have reported the involvement of the Jak–Stat signalling axis as an upstream pathway of the PI3K‐Akt pathway, suggesting its role in M2 macrophage polarisation [31]. In line with these findings, our GSEA analysis showed that this pathway was activated in the Ctrl group compared to the BTX group, indicating its suppression after masseter muscle atrophy (Figure S4c). To further investigate this pathway, the mRNA expression levels of key genes, including *Jak1, Stat6, Klf4* and *Pparγ*, were examined. These genes exhibited increased expression in the BTX + ES group compared to the BTX group (Figure S4c). Furthermore, the protein expression levels of p‐Jak1, p‐Stat6 and their downstream factors Klf4 and Pparγ displayed similar trends, suggesting the potential involvement of the Jak‐Stat6‐Klf4/Pparγ signalling axis in the process of M2 polarisation during the recovery of the atrophic masseter through ES (Figure S4c).

### 3.4 Depletion of Macrophages Inhibits the Restorative Effect of ES on Atrophic Masseter

**FIGURE 5:**
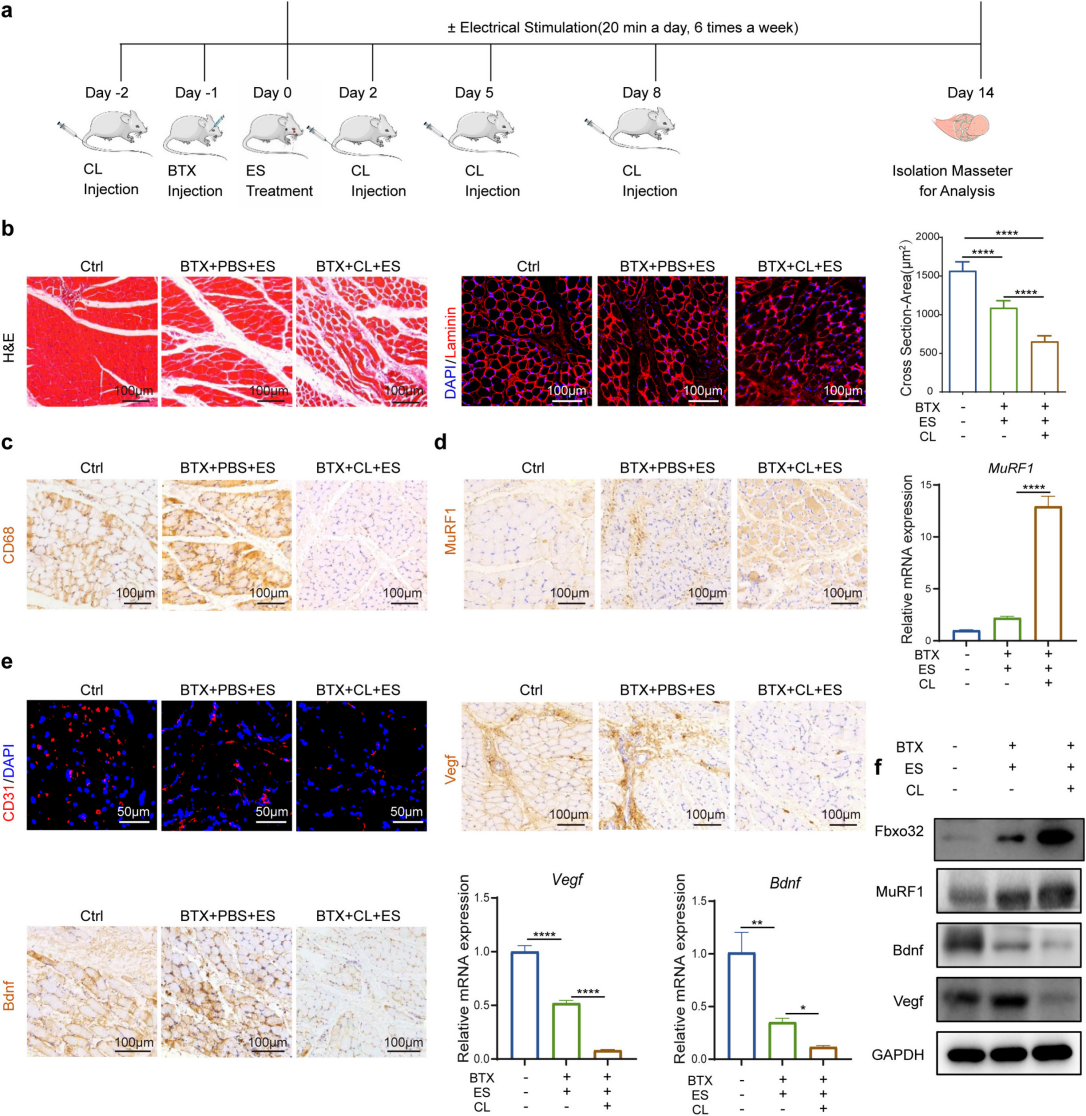
Depletion of macrophages inhibits the restorative effect of ES on atrophic masseter. (a) Experimental design and timeline of CL injection (depletion of macrophages), BTX injection, ES treatment and analysis in rats. (b) H&E stain of cross sections of masseters stained. Scale bars, 100 μm. Representative IF images of laminin (red). Scale bars, 100 μm. Cross‐sectional areas of muscle fibres. (c) Representative IHC images of CD68 (brown). Scale bars, 100 μm. (d) Representative IHC images of MuRF1 (brown). Scale bars, 100 μm; qRT–PCR analysis of MuRF1. (e) Representative IF images of CD31 (red) and DAPI (blue) staining. Scale bars, 50 μm. Representative IHC images of Vegf (brown). Scale bars, 100 μm. Representative IHC images of Bdnf (brown). Scale bars, 100 μm; qRT–PCR analysis of *Vegf* and *Bdnf* mRNA. **f** WB analysis of Fbxo32, MuRF1, Bdnf and Vegf. All data represent three independent experiments (*n* = 3). Error bars indicate SD; *p < 0.05, **p < 0.01, ***p < 0.001, ****p < 0.0001.

Although the role of macrophages in atrophic muscles has been extensively studied, their direct impact on the masseter during ES treatment remains unclear. To directly investigate the role of macrophages in masseter regeneration, we depleted macrophages by injecting clodronate liposomes (CL) into rats (Figure 5a). Animals treated with the PBS exhibited a significantly increased cross‐sectional area of muscle cells compared to the group treated with CL after BTX injection under ES conditions (Figure 5b). Moreover, the number of CD68^+^ cells, indicating macrophages, was significantly reduced after CL injection, confirming the results of macrophage depletion (Figure 5c). Furthermore, we found that the expression of MuRF1, either mRNA or protein, increased in the BTX + CL + ES group, suggesting that macrophages were crucial in promoting muscle regeneration and recovery through ES treatment (Figure 5d). We also investigated the effect of macrophages on angiogenesis and neurogenesis during ES treatment. It revealed that macrophage depletion led to reduced Vegf, CD31 and Bdnf expression in the BTX + CL + ES group in IHC and IF assays. Additionally, mRNA expression analysis of *MuRF1*, *Vegf* and *Bdnf* showed trends consistent with their protein levels (Figure 5d,e; S5a), which aligns with the results obtained from IF and western blot analyses (Figure 5f).

### 3.5 ES Shifts M1 to M2 Macrophages and Promotes Myogenic Cell Proliferation and Differentiation in Vitro

**FIGURE 6:**
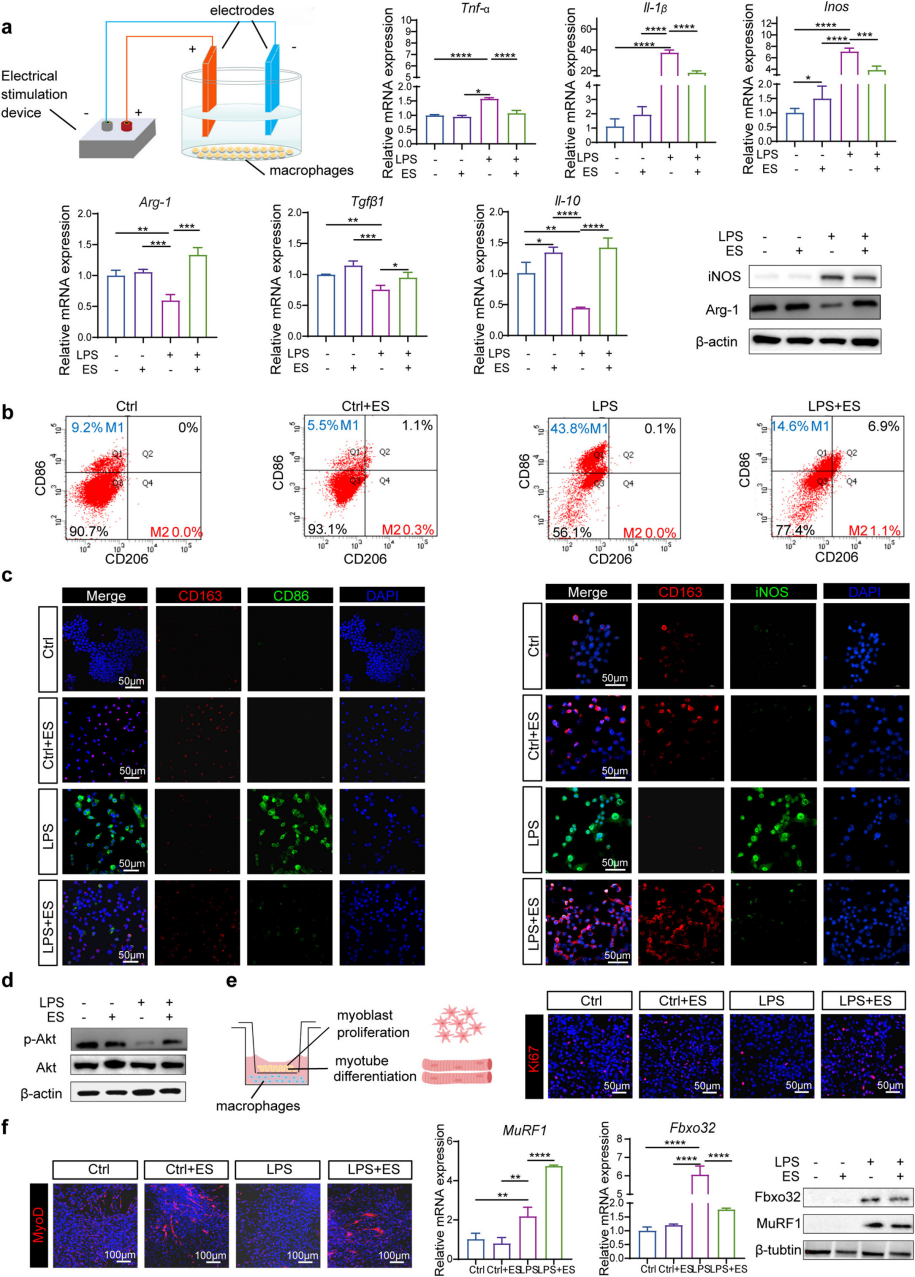
ES shifts M1 to M2 macrophages and promotes myogenic cell proliferation and differentiation in vitro. (a) Diagram of ES on macrophages. Direct current was used to stimulate M1 macrophages induced by LPS; qRT–PCR analysis of *Tnf‐α* and *Inos*. (c) qRT–PCR analysis of *IL‐1β*, *Arg‐ 1*, *Tgfβ1* and *Il‐10*; WB analysis of iNOS and Arg‐1. (b) Flow cytometry of CD86 and CD206. (c) Representative IF images of CD163 (red), CD86 (green) and DAPI (blue). Representative IF images of CD163 (red), iNOS (green) and DAPI (blue). Scale bars, 50 μm. d WB analysis of p‐AKT and AKT. (e) Diagram of macrophage co‐culture with myoblast and myotube. Representative IF images of Ki67 (red) and DAPI (blue). Scale bars, 50 μm. (f) Representative IF images of MyoD (red) and DAPI (blue). Scale bars, 100 μm; qRT–PCR analysis of ***MuRF1*** and *Fbxo32* mRNA; WB analysis of MuRF1 and Fbxo32. All data represent three independent experiments (*n* = 3). Error bars indicate SD; *p < 0.05, **p < 0.01, ***p < 0.001, ****p < 0.0001.

Recent research demonstrates that macrophage polarisation plays a crucial role in muscle regeneration by modulating myoblast proliferation and differentiation [32]. To further investigate the direct effects of ES on macrophage polarisation, we applied direct current stimulation to M1 macrophages and co‐cultured the induced macrophages with myogenic cells to assess the changes from the aspects of cell proliferation and differentiation by Cellular ES instrument. Firstly, we used LPS to simulate the inflammatory environment [26]. After LPS induction, RAW264.7 cells polarised to the M1 phenotype, with the increased mRNA levels of inflammatory markers *Tnf‐α*, *Il‐1β* and *Inos*. After ES treatment, the expression of these genes decreased while the expression of M2 marker genes such as *Arg‐1, Il‐10* and *Tgfβ*1 increased. Subsequently, it was found that after LPS induction, the protein expression of iNOS increased. But following ES, it decreased, while the protein expression of Arg‐1 increased (Figure 6a; S5b). Flow cytometry results exhibited a similar trend; CD86, the surface marker of M1 macrophages, increased after LPS stimulation but decreased following ES, while the surface marker CD206 of M2 macrophages decreased after LPS stimulation but increased following ES. To further observe these changes, we performed cellular IF staining. It showed an increase in CD86^+^ and iNOS^+^ cells (Figure 6b) following LPS induction, which decreased after ES. Additionally, M2 polarisation occurred, indicated by an increase in CD163^+^ cells after ES (Figure 6c).

As mentioned before, ES promotes M2 polarisation of macrophages through the activation of the PI3K‐Akt pathway in vivo. Therefore, we next investigated whether PI3K‐ Akt pathway plays an important role in the LPS‐induced polarisation after ES in vitro. It showed that the p‐Akt expression level increased after ES compared to that in the LPS group, indicating PI3K‐Akt pathway activation, consistent with the results in vivo findings (Figure 6d; S5b).

Subsequently, we further investigated the effects of macrophages on myoblasts by co‐culturing them with myoblasts and differentiated myotubes. IF results showed that the Ki67 expression in the LPS group was reduced compared to the Ctrl group, indicating that M1 macrophages inhibit the proliferation of myoblasts (Figure 6e). However, its expression in the LPS + ES group showed a certain degree of increase. Furthermore, after co‐culturing with differentiated myotubes, the LPS group inhibited myotube differentiation compared to the Ctrl group, resulting in decreased MyoD expression and induced myotube atrophy, with increased expression of MuRF1 and Fbxo32. However, after ES treatment, MyoD expression showed some increase compared to the LPS group, and the expression of MuRF1 and Fbxo32 decreased (Figure 6f; S5b). These results indicate that ES induces M2 polarisation of macrophages and promotes the proliferation and differentiation of myoblasts, leading to a certain degree of recovery in atrophied myotubes.

**FIGURE 7:**
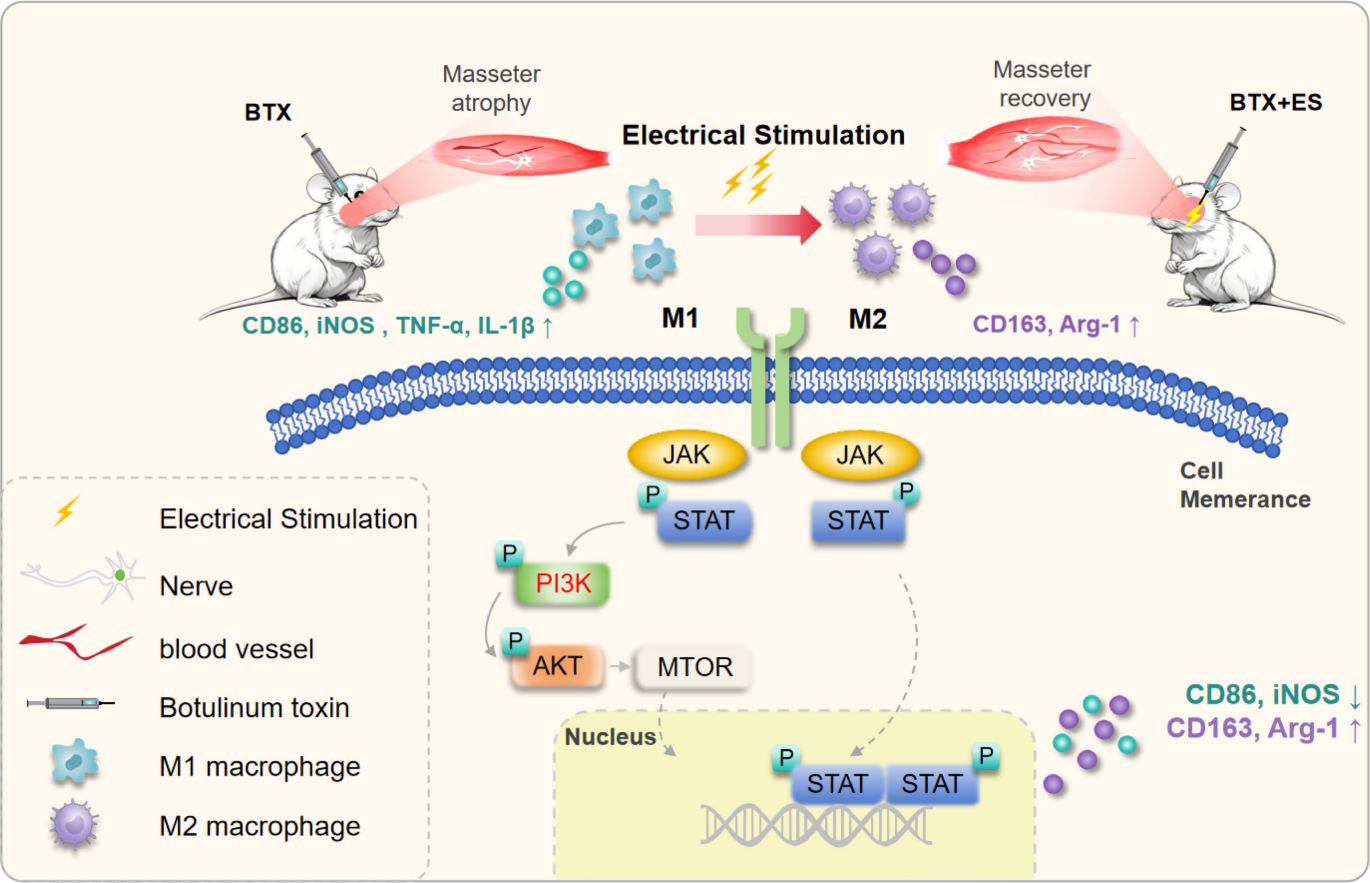
Schematic diagram of this study.

These findings indicate that macrophages may play a crucial role in the recovery of atrophic muscles during ES treatment (Figure 7).

## 4 Discussion

In our investigation, we have illustrated that ES treatment facilitates the recovery of atrophic masseter muscle, promoting angiogenesis and neurogenesis. Notably, this recuperative process is associated with a shift in macrophage phenotype from a pro‐inflammatory M1 state to an anti‐inflammatory M2 state.

Lotri‐Koffi et al. previously reported that chronic neuromuscular ES improved cardiometabolic muscle mass and insulin sensitivity in a mouse model [33]. Similarly, early cyclical neuromuscular ES therapy was found to enhance muscle strength and induce hypertrophy following incomplete spinal cord injury in a rat model [34]. Furthermore, Bao et al. recently demonstrated that ES treatment of the gastrocnemius and tibialis anterior muscles in ICU patients led to improvements in muscle strength, joint range of motion and cross‐sectional muscle area of the lower leg [35]. These reports suggest a beneficial role for ES activation in muscle regeneration, but there is limited research on the effects of ES on the masseter. Given this background, we investigated whether ES could prevent masseter muscle atrophy. The effects of ES appear to vary depending on factors such as intensity and frequency. Most studies have used ES frequencies between 10 and 50 Hz and voltages between 10 and 30 V [10, 28, 36]. Consistent with these findings, our results confirm the effectiveness of both 10 and 50 Hz frequencies in our study. However, some studies suggested that low‐frequency stimulation may yield better results [37], possibly due to higher frequencies causing muscle stiffness or fatigue. In our pre‐experiment, we found that stimulation intensities exceeding 7 mA led to strong resistance and pain responses in rats, regardless of frequency. We observed significant recovery of masseter muscles with stimulation current at 6–7 mA. However, there is a paucity of research on the optimal current range for ES applications.

Our study confirmed that the recovery of atrophied muscles is accompanied by the regeneration of blood vessels and nerves. Previous studies showed some similar views. For angiogenesis, Nakagawa et al. [29] performed ES on the tibialis anterior muscle following denervation and showed the beneficial effects of ES in reducing muscle atrophy and capillary regression in the early stages of denervation‐induced muscle disuse in rat hind limbs. Regarding neurogenesis, Willand et al. [38] implanted intramuscular electrodes into gastrocnemius muscles in which the tibial nerve was transected and immediately repaired. They observed a significant upregulation of glial cell line‐derived neurotrophic factor (Bdnf and GDNF) mRNA levels in muscles subjected to daily ES compared to those without stimulation. Liao et al. [39] also suggested that ES could promote regrowth and upregulate electrophysiological function in Taxol‐treated peripheral nerve injury.

As a potential link between ES and regenerative immune responses, this study found that ES led to a phenotypic switch from an M1 to an M2 macrophage in the process of muscle repair. Previous histological observations have shown that initially, macrophages predominantly exhibit an M1 activation state, involved in pathogen and cell debris clearance. The peak presence of phagocytic M1 macrophages is observed at 2 days post‐injury, followed by a transition to non‐phagocytic M2 macrophages, which aid in inflammation resolution and tissue remodelling, peaking around 4–7 days post‐injury [18]. Previously, Shimada et al. [20] found that M1 macrophage exacerbated muscle atrophy, and inhibition of M1 reversed this process. Hu et al. [40] reported that the expression of the M2 marker Arg‐1 increased 2 days after the initiation of ES and remained at a high level at Day 3, which is similar to our results. Additionally, they also found a parallel change in IGF‐1 and concluded that ES ameliorated skeletal muscle atrophy by enhancing the IGF‐1 signalling pathway.

In our study, we demonstrated that ES activates PI3K/Akt in masticatory muscle macrophages, promoting M2 polarisation and muscle repair. While the exact molecular mechanisms by which ES triggers PI3K/Akt activation in macrophages remain to be fully elucidated, several lines of evidence offer plausible explanations. Emerging evidence suggests that electrical signals may modulate intracellular calcium dynamics or membrane potential changes, thereby initiating downstream signalling cascades involving receptor tyrosine kinases (RTKs) or integrin‐mediated pathways [41, 42]. For instance, studies have shown that ES can induce mechano‐transduction via ion channel activation or piezoelectric effects, leading to PI3K/Akt pathway activation in immune cells. Specifically, in macrophages, ES may mimic endogenous electrogenic cues (e.g., surface potential gradients) that regulate PI3K/Akt signalling to promote M2 polarisation, as observed in biomaterial‐mediated ES models [42, 43, 44]. While the exact upstream triggers in our system require further investigation, these findings provide plausible mechanistic frameworks for future studies. Overall, our results indicate that ES facilitates the regeneration of atrophied masseter muscles, potentially through mechanisms such as angiogenesis and nerve regeneration, with the phenotypic shift of macrophages likely playing a role in this process. However, there are some limitations. First, the contraction of the right masseter muscle caused by ES therapy may influence chewing patterns and the left masseter, which may change the occlusal load on the alveolar bone. Although we have published that ES could prevent condyle and subchondral degeneration in the temporomandibular joint following the masseter atrophy [45], it remains unclear whether the bone remodels to adapt to the changes in the masticatory muscles. Secondly, due to species differences, the morphology and function of the masticatory muscles in rats are likely to be different from those in large animals or humans. Therefore, more exploration is required in future experiments involving large animals. Besides, a limitation of this study is the absence of validation using denervation or stroke‐induced masticatory muscle atrophy models. These models may reveal distinct atrophy mechanisms, and future studies will incorporate them to broaden the applicability and mechanistic insights of our findings. In addition, the roles of other immune cells and the origins of infiltrating M1 macrophages within the atrophied masseter muscle still need additional study. Further research is essential to fully understand the role of macrophages in the regeneration of atrophied masseter muscles and the potential impact of ES therapy on chewing patterns and occlusal load. Additionally, the distinctions in masticatory muscles across species and the potential contribution of other immune cells to the regeneration process necessitate further investigation.

## Conflicts of Interest

The authors declare no conflicts of interest.

## Supporting information


**Figure S1** Electrical stimulation device. (a) BTX injection site in superficial (S) and deep (D) masseter muscles and photograph of the ES device. (b) Photograph of masseter muscles and muscle weight was measured. (c) Body weight was measured. (d) H&E stain of cross sections of masseter muscles with different ES parameters (top). Scale bars, 100 μm. Representative IF images of laminin (bottom). Scale bars, 50 μm. (e) Cross‐sectional areas of muscle fibres. (f) Quantification of relative band intensity of MuRF1, Fbxo32 and Vegf and Bdnf. All data represent three independent experiments (*n* = 3). Error bars indicate SD; *p < 0.05, **p < 0.01, ***p < 0.001, ****p < 0.0001.
Figure S2 (a–b) qRT–PCR analysis of *Tnf‐α* and *IL‐1β* messenger RNA. (c) Representative IF images of TUNEL (green), Laminin (red) and DAPI (blue) staining. Scale bars, 50 μm. All data represent three independent experiments (*n* = 3). Error bars indicate SD; *p < 0.05, **p < 0.01, ***p < 0.001, ****p < 0.0001.
Figure S3 Single‐nucleus sequencing analysis. (a) UMAP plots of 31 clusters of all cells. (b) UMAP plots of 7 clusters of macrophages and T‐B cells. (c) Pseudo‐time analysis of gene expression of *Cd163*, *Cd74*, *F13a1*, *Frmd4b*, *Mrc1*, *Tnnc2*, *Cfh*, *Kcnt2*, *Myh4*, *Ptprc*, *Slc9a9* and *Wnk2*. (d) Cell‐chat between M2 macrophages with the other cells. (e) Quantification of relative band intensity of CD163, CD86, iNOS and Arg‐1. (f) Quantification of relative band intensity of CD163, CD86, iNOS, and Arg‐1. All data represent three independent experiments (*n* = 3). Error bars indicate SD; *p < 0.05, **p < 0.01, ***p < 0.001, ****p < 0.0001.
Figure S4 ES promotes the transition from M1 to M2 macrophages through the Jak–Stat6 pathway. (a) Quantification of relative band intensity of p‐PI3K, PI3K, CD163, CD86 and iNOS. (b) Quantification of relative band intensity of Vegf, MuRF1, Fbxo32 and Bdnf. (c) GSEA analysis of Jak–Stat pathway in the control group compared to the BTX group; qRT–PCR analysis of *Jak‐1*, *Stat6*, *Klf4* and *Ppar‐γ* messenger RNA; p‐Jak1, Jak‐1, p‐Stat6, Stat6, Klf4 and Ppar‐γ protein expression and quantification of relative band intensity. All data represent three independent experiments (*n* = 3). Error bars indicate SD; *p < 0.05; **p < 0.01; ***p < 0.001; ****p < 0.0001.
Figure S5 (a) Quantification of relative band intensity of Fbxo32, MuRF1, Vegf and Bdnf. (b) Quantification of relative band intensity of iNOS, Arg‐1, p‐Akt, Akt, MuRF1 and Fbxo32. All data represent three independent experiments (*n* = 3). Error bars indicate SD; *p < 0.05; **p < 0.01; ***p < 0.001; ****p < 0.0001.
Table 1 Primer sequence for genes.
Table 2 Specific antibody catalogue list.
