# Supplementary material for: Atrophy Masseter Recovery by Electrical Stimulation Mediated M2‐Like Macrophage Polarisation via JAK/PI3K/AKT Pathway
Source: J Cachexia Sarcopenia Muscle. 2025 Aug 15;16(4):e70048. doi: 10.1002/jcsm.70048 (PMC12356994; doi:10.1002/jcsm.70048)
Supplement: Supplementary file 1 — Figure S1 Electrical stimulation device. (a) BTX injection site in superficial (S) and deep (D) masseter muscles and photograph of the ES device. (b) Photograph of masseter muscles and muscle weight was measured. (c) Body weight was measured. (d) H&E stain of cross sections of masseter muscles with different ES parameters (top). Scale bars, 100 μm. Representative IF images of laminin (bottom). Scale bars, 50 μm. (e) Cross‐sectional areas of muscle fibres. (f) Quantification of relative band intensity of MuRF1, Fbxo32 and Vegf and Bdnf. All data represent three independent experiments (n = 3). Error bars indicate SD; *p < 0.05, **p < 0.01, ***p < 0.001, ****p < 0.0001. Figure S2 (a–b) qRT–PCR analysis of Tnf‐α and IL‐1β messenger RNA. (c) Representative IF images of TUNEL (green), Laminin (red) and DAPI (blue) staining. Scale bars, 50 μm. All data represent three independent experiments (n = 3). Error bars indicate SD; *p < 0.05, **p < 0.01, ***p < 0.001, ****p < 0.0001. Figure S3 Single‐nucleus sequencing analysis. (a) UMAP plots of 31 clusters of all cells. (b) UMAP plots of 7 clusters of macrophages and T‐B cells. (c) Pseudo‐time analysis of gene expression of Cd163, Cd74, F13a1, Frmd4b, Mrc1, Tnnc2, Cfh, Kcnt2, Myh4, Ptprc, Slc9a9 and Wnk2. (d) Cell‐chat between M2 macrophages with the other cells. (e) Quantification of relative band intensity of CD163, CD86, iNOS and Arg‐1. (f) Quantification of relative band intensity of CD163, CD86, iNOS, and Arg‐1. All data represent three independent experiments (n = 3). Error bars indicate SD; *p < 0.05, **p < 0.01, ***p < 0.001, ****p < 0.0001. Figure S4 ES promotes the transition from M1 to M2 macrophages through the Jak–Stat6 pathway. (a) Quantification of relative band intensity of p‐PI3K, PI3K, CD163, CD86 and iNOS. (b) Quantification of relative band intensity of Vegf, MuRF1, Fbxo32 and Bdnf. (c) GSEA analysis of Jak–Stat pathway in the control group compared to the BTX group; qRT–PCR analysis of Jak‐1, Stat6, Klf4 an [file JCSM-16-e70048-s001.docx]

**Atrophy masseter recovery by electrical stimulation mediated M2-like macrophage polarization**

This document includes:

Supplement methods

Supplement Figure S1 to Figure S5.

Supplement Table 1.

Supplement Table 2.

**Supplement methods**

**Histology**

Masseter tissue samples were fixed in 4% paraformaldehyde (PFA) for 24-48 hours, washed, dehydrated with graded ethanol, and embedded in paraffin. The samples were cut into 4 μm thick sections (Leica, USA). To compare histopathology between lesions, we harvested muscle near the center of the injection. Then, sections were stained with hematoxylin-eosin (H&E) (BASO, China). To measure muscle fiber size, cross-sections of muscle fibers in the abdominal region were quantified by ImageJ software.

**Immunohistochemistry staining**

For IHC and IF staining in vivo samples, slides were deparaffinized in xylene and graded alcohol first, followed by heat-induced antigen retrieval in a microwave oven with sodium citrate buffer (ZSGB-Bio, China). After that, sections were treated with 3% H_2_O_2_ for 15 minutes to block catalase and then blocked with 5% goat serum (Bio sharp, China) for 30 minutes at room temperature. For IF staining of cell samples, cells were fixed in 4% paraformaldehyde (PFA) for 30min, followed by permeabilized with 0.3% Triton X-100-PBS for 20min, then blocked with 5% goat serum (Bio sharp, China) for 30 minutes at room temperature. To analyze the amount of muscle atrophy, the slides were incubated with anti-MuRF1 (Santa Cruz Biochemicals, USA). Angiogenesis and neurogenesis were identified by staining with antibodies against Vegf (Huabio, China), CD31(ABclonal, China), and Bdnf (Huabio, China). IF analysis was performed to determine the types of macrophages in the masseter. Sections were incubated with CD86 (Huabio, China) or CD163 antibodies (Huabio, China) overnight at 4 °C. To analyze the proliferation and differential of myoblast，cells were incubated with Mki67 (Abcam, the USA) or Myod antibodies (Abcam, the USA) overnight at 4 °C. The next day, for IHC, slides were incubated with HRP-conjugated secondary antibodies (Huabio, China) for 1h, developed with 3,3'-diaminobenzidine (DAB, ZSGB-Bio, China) and counterstained with Mayer's hematoxylin. For IF, sections and cells were incubated with Alexa Fluor 488- and Alexa Flour 647-conjugated secondary antibodies (Huabio, China) for 1 h and then stained with DAPI (Beyotime, China) for 5 min. The positive area of IHC and MFI of IF were quantified by ImageJ software.

**Western blotting**

The masseter muscle samples were triturated in tubes containing protease inhibitors in RIPA buffer (Beyotime, China) to extract proteins. After quantitative measurement of protein concentration by BCA assay (Beyotime, China). Then, the proteins (10 µg) were loaded on sodium dodecyl sulfate-polyacrylamide (SDS) gels (YaMei, China) and transferred to polyvinylidene fluoride (PVDF) membranes (Millipore, USA). After that, the membranes were blocked with 5% BSA nonfat dry milk in TBS-T for 1 h at room temperature and then incubated with the primary antibody overnight at 4 °C [GAPDH (Zenbio, China), MuRF1 (Santa Cruz), Fbxo32 (HUABIO), iNOS (Affinity, China), Arg-1 (Affinity), CD86 (Huabio), CD31 (Proteintech), CD163 (Huabio), Vegf (Affinity), Bdnf (Huabio), Jak1 (Affinity), p-Jak1 (Affinity), Stat6 (Proteintech), p-Stat6 (Affinity), Klf4 (Huabio), Ppar γ(Proteintech), Akt (Proteintech, China), p-Akt (Proteintech)]. The next day, the membranes were incubated with peroxidase-conjugated secondary antibodies for 1 h at room temperature and then washed with TBS-T. Finally, the proteins in the membranes were detected using enhanced chemiluminescent ECL detection reagents (Zenbio, China) by an imaging system (Tanon, China) and quantified by ImageJ software. **Specific antibody catalog is listed in Supplement Table 2.**

**RNA extraction and qPCR**

Total RNA was extracted from masseter muscle tissue using TRIzol reagent (Ambion, Thermofisher, America). Then, complementary DNA (cDNA) was synthesized using the Prime Script RT Master Mix assay (TaKaRa Bio, Otsu, Japan). qPCR was performed on a real-time system (Applied Biosystems, Carlsbad, CA, USA) using the SYBR Premix Ex Taq II kit (TaKaRa, Japan). Glyceraldehyde-3-phosphate dehydrogenase (Gapdh) was used as a reference gene, and the relative expression levels of individual genes were calculated using the ^∆∆^CT method. The primer sequences involved in this study are listed in supplemental Table 1.

**C2C12**

C2C12 cells were cultured in Dulbecco’s Modified Eagle Medium (DMEM, D5546) supplemented with 10% (v/v) FBS, 1% (v/v) Penicillin/Streptomycin (Sigma-Aldrich, Madrid, Spain). The cells were kept at 37℃ in a humidified incubator with 5% CO_2_. To determine the myogenic differentiation potential of primary human myoblasts, cells were treated with differentiation media (DMEM, 2% horse serum).

**Cell co-culture**

Trans well chamber was used to co-culture macrophages with C2C12, with 5 × 10^4^ C2C12 seeded in the upper chamber and 1 × 10^5^ RAW264.7 cells in the lower chamber.

**Flow cytometry**

Single-cell suspensions of RAW264.7 (2-10x10^6^) were pre-incubated with 1 μg of anti-CD16/32 (BioLegend) to block Fc receptors, followed by surface staining with PE anti-CD86 (BioLegend) and APC anti-CD206 (BioLegend) followed the standard protocols. All samples were analyzed on a CytoFLEX flow cytometer (Beckman) with CytExpert software.

**Supplement Figure**


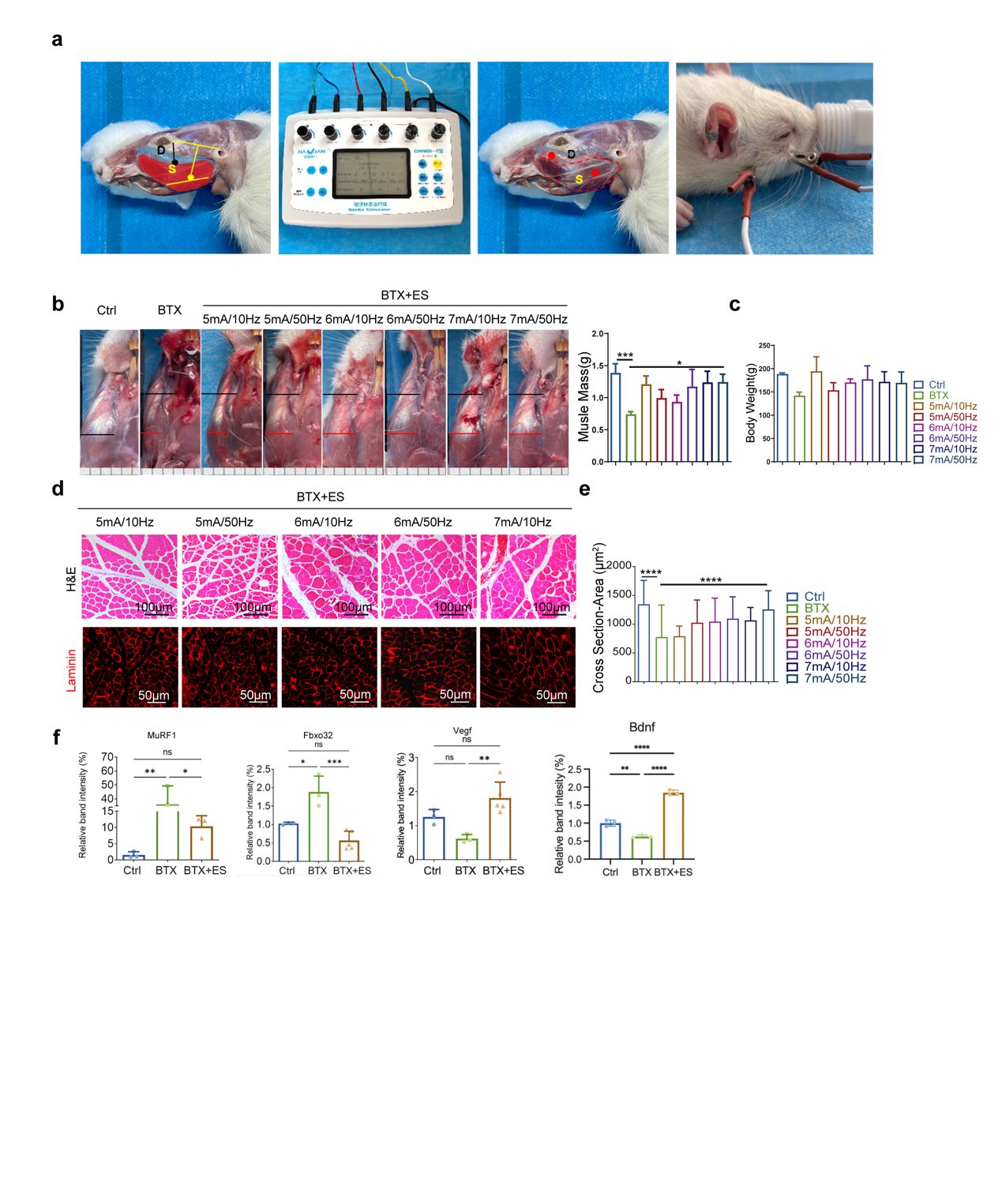


**Figure S1 Electrical stimulation device. a** BTX injection site in superficial (S) and deep (D) masseter muscles and photograph of the ES device. **b** Photograph of masseter muscles and muscle weight was measured. **c** Body weight was measured. **d** H&E stain of cross sections of masseter muscles with different ES parameters (top). Scale bars, 100 μm; Representative IF images of laminin (bottom). Scale bars, 50 μm. **e** Cross-sectional areas of muscle fibers. **f** Quantification of relative band intensity of MuRF1, Fbxo32, Vegf and Bdnf. All data represent three independent experiments (n=3). Error bars indicate SD, *P < 0.05, **P < 0.01, ***P < 0.001, ****P < 0.0001.

**
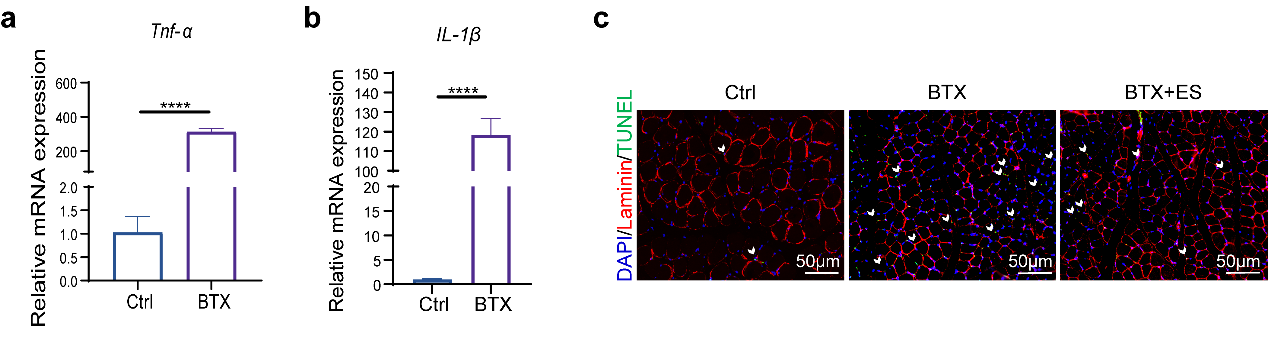
 Figure S2** **(a-b)** qRT–PCR analysis of *Tnf-α* and *IL-1β* messenger RNA. **c** Representative IF images of TUNEL (green), Laminin (red), and DAPI (blue) staining. Scale bars, 50 μm. All data represent three independent experiments (n=3). Error bars indicate SD, *P < 0.05, **P < 0.01, ***P < 0.001, ****P < 0.0001.


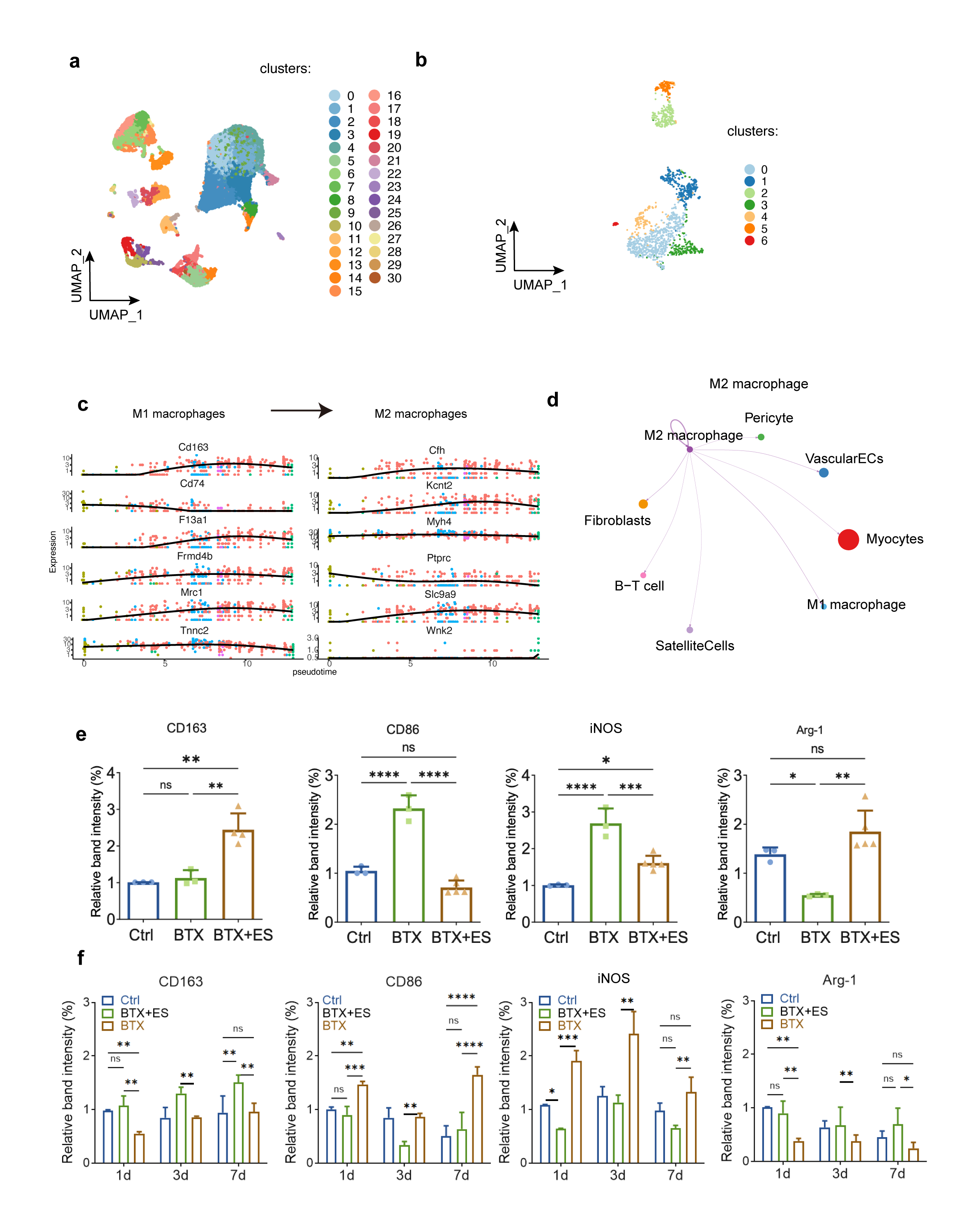


**Figure S3** **Single-nucleus sequencing analysis. a** UMAP plots of 31 clusters of all cells. **b** UMAP plots of 7 clusters of macrophages and T-B cells. **c** Pseudo-time analysis of gene expression of *Cd163, Cd74, F13a1, Frmd4b, Mrc1, Tnnc2, Cfh, Kcnt2, Myh4, Ptprc, Slc9a9,* and *Wnk2*. **d** Cell-chat between M2 macrophages with the other cells. **e** Quantification of relative band intensity of CD163, CD86, iNOS, and Arg-1. **f** Quantification of relative band intensity of CD163, CD86, iNOS, and Arg-1. All data represent three independent experiments (n=3). Error bars indicate SD, *P < 0.05, **P < 0.01, ***P < 0.001, ****P < 0.0001.


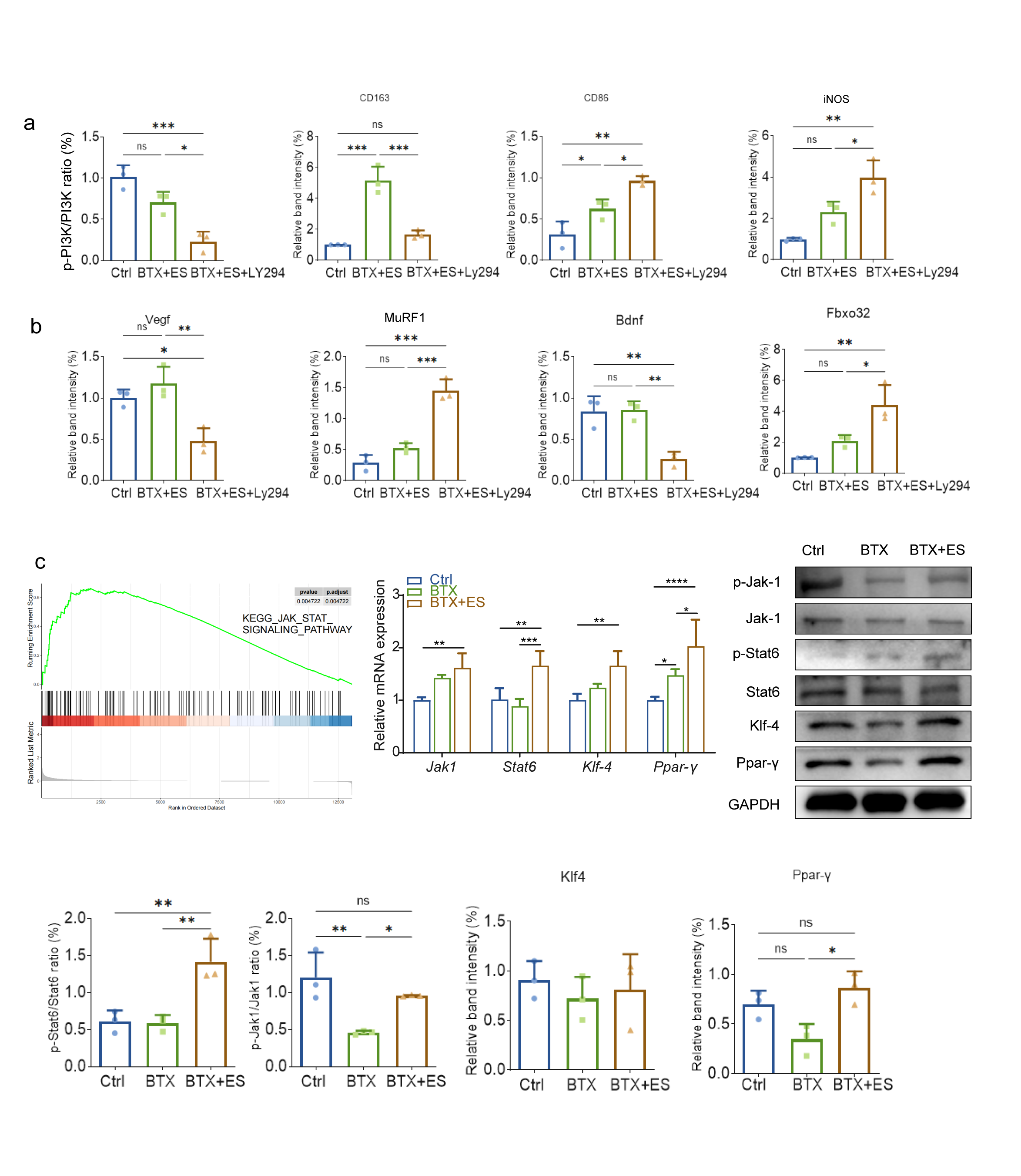


**Figure S4** **ES promotes the transition from M1 to M2 macrophages through the Jak-Stat6 pathway**. **a** Quantification of relative band intensity of p-PI3K, PI3K, CD163, CD86, and iNOS. **b** Quantification of relative band intensity of Vegf, MuRF1, Fbxo32, Bdnf. **c** GSEA analysis of Jak-Stat pathway in the control group compared to the BTX group; qRT–PCR analysis of *Jak-1*, *Stat6*, *Klf4*, and *Ppar-γ* messenger RNA; p-Jak1, Jak-1, p-Stat6, Stat6, Klf4, and Ppar-γ protein expression and quantification of relative band intensity. All data represent three independent experiments (n=3). Error bars indicate SD, *P < 0.05, **P < 0.01, ***P < 0.001, ****P < 0.0001.


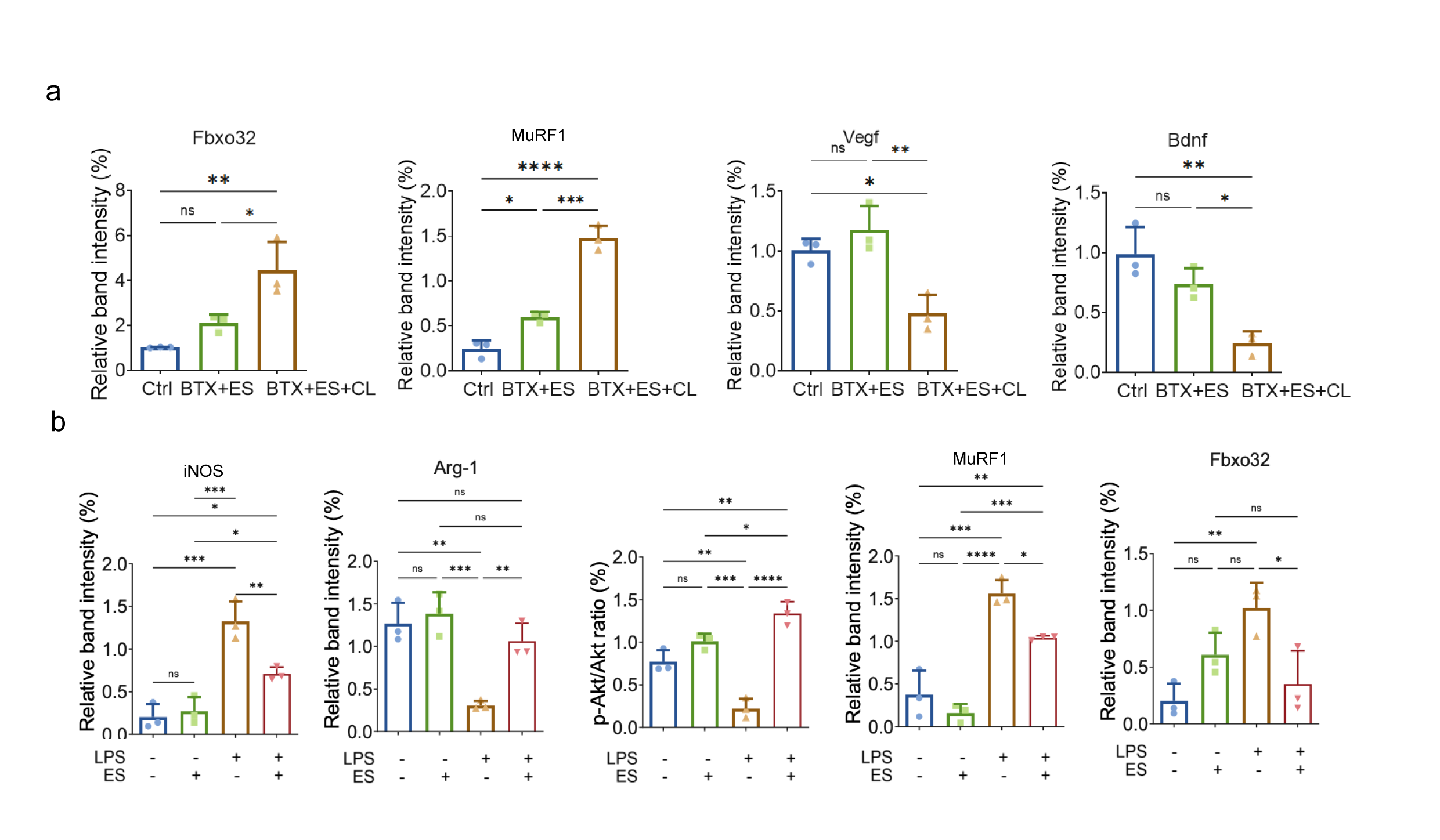


**Figure S5** **a** Quantification of relative band intensity of Fbxo32, MuRF1, Vegf, and Bdnf. **b** Quantification of relative band intensity of iNOS, Arg-1, p-Akt, Akt, MuRF1, and Fbxo32. All data represent three independent experiments (n=3). Error bars indicate SD, *P < 0.05, **P < 0.01, ***P < 0.001, ****P < 0.0001.

**Supplement Table**

| Gene | Sequence |
| --- | --- |
| *MuRF1* | Forword-GGTGCCTACTTGCTCCTTGTGC  Reverse-GCTCAGTTCAGTCTTCTGTCCTTGG |
| *Fbxo32* | Forword-ACTCATACGGGAACTTCTCCAGACC  Reverse-GCTGCTGTTGCCAGTGTAGAGTG |
| *Vegf* | Forword-CACCAAAGCCAGCACATAGGAGAG  Reverse-CTGCGGATCTTGGACAAACAAATGC |
| *Bdnf* | Forword-TGGAACTCGCAATGCCGAACTAC  Reverse-TCCTTATGAACCGCCAGCCAATTC |
| *Inos* | Forword-TCTTGGAGCGAGTTGTGGATTGTTC  Reverse-AGTGATGTCCAGGAAGTAGGTGAGG |
| *CD86* | Forword-GCTGTCTCTTTCTGCTGGTCGTC  Reverse-CTCACAAGTCTTTCTGCTGGGTCTG |
| *Arg-1* | Forword-AGACCACAGTATGGCAATTGGAAGC  Reverse-TTGTCAGCGGAGTGTTGATGTCAG |
| *CD163* | Forword-TTAGAATCACAGCATGGCACAGGTC  Reverse-CCACAAGAGGAAGGCAATGAGAAGG |
| *Gapdh* | Forword-ACAGCAACAGGGTGGTGGAC  Reverse-TTTGAGGGTGCAGCGAACTT |
| *Stat6* | Forword-GTCTGGATGAAGTCCTGCGAACC  Reverse-AGTGGAGGCTTGGCTGAGGTC |
| *Klf-4* | Forword-GGACGGCTGTGGGTGGAAATTC  Reverse-CCTGTCGCACTTCTGGCACTG |
| *Pparg* | Forword-TCTGTGGACCTCTCTGTGATGGATG  Reverse-AGGCTCTACTTTGATCGCACTTTGG |
| *Akt1* | Forword-GGCAGGAGGAGGAGACGATGG  Reverse-GCAGGACACGGTTCTCAGTAAGC |
| *IL-10* | Forword-ACTGCTATGTTGCCTGCTCTTACTG  Reverse-TGGGTCTGGCTGACTGGGAAG |
| *Tnf-α* | Forword-CACCACGCTCTTCTGTCTACTGAAC  Reverse-TGGGCTACGGGCTTGTCACTC |
| *Jak1* | Forword-CCTGGCTACCTTGGAAACCTTAAC  Reverse-CGTTGGAATGGCACCGACTC |
| *Pi3k* | Forword-GATAGACCACCGCTTCCTCCTC  Reverse-TGCCCTGTTCCTCTGCCTTC |
| *Mtor* | Forword-CATCCAGAGATACGCCGTCATTCC  Reverse-TCAGAGTCAGGTGGTCATAGTCAGG |

**Table 1** Primer sequence for genes.

| MuRF1 | Santa Cruz | Cat # sc-398608 |
| --- | --- | --- |
| Fbxo32 | HUABIO | Cat # ET7109-25 |
| Vegf | Affinity | Cat # AF5131 |
| Bdnf | HUABIO | Cat # ER130915 |
| CD31 | ABclonal | Cat # A19014 |
| CD163 | HUABIO | Cat # ER1804-03 |
| CD86 | HUABIO | Cat # ER1906-01 |
| Arg-1 | Affinity | Cat # DF6657 |
| iNOS | Affinity | Cat # AF0199 |
| Pi3k | Affinity | Cat # AF6241 |
| p-Pi3k | Affinity | Cat # AF3242 |
| Akt | Proteintech | Cat # 10176-2-AP |
| p-Akt | Proteintech | Cat # 66444-1-Ig |
| MyoD | Abcam | Cat # ab307805 |
| CD68 | HUABIO | Cat # ER1901-32 |
| p-Stat6 | Affinity | Cat # AF3301 |
| Stat6 | Proteintech | Cat # 51073-1-AP |
| JAK1 | Affinity | Cat # AF5012 |
| p-JAK1 | Affinity | Cat # AF2012 |
| Klf4 | HUABIO | Cat # ET1702-71 |
| Ppar gamma | Proteintech | Cat # 16643-1-AP |

**Table 2** Specific antibody catalog list.
